# Evidence for Snake Venom Plasticity in a Long-Term Study with Individual Captive *Bothrops atrox*

**DOI:** 10.3390/toxins11050294

**Published:** 2019-05-24

**Authors:** Diana R. Amazonas, Luciana A. Freitas-de-Sousa, Daniele P. Orefice, Leijiane F. de Sousa, Melissa G. Martinez, Rosa H. V. Mourão, Hipócrates M. Chalkidis, Plínio B. Camargo, Ana M. Moura-da-Silva

**Affiliations:** 1 Laboratório de Imunopatologia, Instituto Butantan, 05503-900 São Paulo, Brazil; diana.amazonas@gmail.com (D.R.A.); luciana.sousa@butantan.gov.br (L.A.F.-d.-S.); daniele.orefice@butantan.gov.br (D.P.O.); leijiane.sousa@butantan.gov.br (L.F.d.S.); 2 Laboratório de Ecologia Isotópica, Centro de Energia Nuclear na Agricultura—USP, 13416-000 Piracicaba, Brazil; martinez.melissa@yahoo.com.br (M.G.M.); pcamargo@cena.usp.br (P.B.C.); 3 Programa de Pós-Graduação em Recursos Naturais da Amazônia, Laboratório de Bioprospecção e Biologia Experimental, Universidade Federal do Oeste do Pará—UFOPA, 68040-070 Santarém, PA, Brazil; mouraorhv@yahoo.com.br; 4 Laboratório de Pesquisas Zoológicas, Unama Centro Universitário da Amazônia, 68010-200 Santarém, PA, Brazil; hchalkidis@gmail.com

**Keywords:** venom variability, metalloproteinases, enzyme toxins

## Abstract

Variability in snake venom composition has been frequently reported and correlated to the adaptability of snakes to environmental conditions. Previous studies report plasticity for the venom phenotype. However, these observations are not conclusive, as the results were based on pooled venoms, which present high individual variability. Here we tested the hypothesis of plasticity by influence of confinement and single diet type in the venom composition of 13 adult specimens of *Bothrops atrox* snakes, maintained under captivity for more than three years. Individual variability in venom composition was observed in samples extracted just after the capture of the snakes. However, composition was conserved in venoms periodically extracted from nine specimens, which presented low variability restricted to the less abundant components. In a second group, composed of four snakes, drastic changes were observed in the venom samples extracted at different periods, mostly related to snake venom metalloproteinases (SVMPs), the core function toxins of *B. atrox* venom, which occurred approximately between 400 and 500 days in captivity. These data show plasticity in the venom phenotype during the lifetime of adult snakes maintained under captive conditions. Causes or functional consequences involved in the phenotype modification require further investigations.

## 1. Introduction

Venoms are considered trophic adaptations that enable venomous snakes to use potent toxins as a chemical means to subdue their prey [1]. Consisting predominantly of proteins, snake venoms include a limited number of protein families [2]; however, each group includes several isoforms that may present distinct functional activities [3]. In *Bothrops* snake venoms, snake venom metalloproteases (SVMPs), snake venom serine proteases (SVSPs), C-type lectin-like toxins (CTLs), and phospholipases A_2_ (PLA_2_s) are the most abundant toxin families and are commonly related to impacts on prey hemostasis [4]. Other toxin families, such as cysteine-rich secretory proteins (CRISPs), L-amino acid oxidases (LAAOs), and other components, are also included in the venom composition, but generally in lower abundance and have not yet been related to serious disturbances in the coagulation system or tissue damage. Venoms from *B. atrox* snakes are composed predominantly of SVMPs, representing approximately 50% of the proteome and more than 70% of transcriptome, with 42 distinct transcripts completely sequenced in studies performed with *B. atrox* from the same areas used in this study [5]. The evolutionary mechanisms of this gene family allowed the structural and functional diversity of SVMPs in viper venoms [6,7]. SVMPs are able to interact with different targets that control hemostasis or other essential physiological functions in prey and predators [3,8]. The action of SVMPs involve mechanisms such as hydrolysis of extracellular matrix components [9], disruption of capillary vessels [10,11], activation or cleavage of coagulation factors [12,13], and activation of several inflammatory cells with the release of pro-inflammatory mediators [8]. Following SVMPs, CTLs are the second most abundant toxin family in *B. atrox* venom (~20%) and PLA_2_s and SVSPs are present in in lower amounts in venom proteome (6–9% and 4–7%, respectively) [5].

Snake venoms present high degrees of variability in composition, which is usually associated as an advantage for the consumption and digestion of a wide range of prey [14,15,16]. The variability in snake venom composition is, in most cases, derived from differences in expression levels of toxin paralogue gene coding for distinct toxin isoforms [5,17]. These genes have been diversified by multiple mechanisms, including several steps of duplication and modifications of the distinct copies by accumulation of substitutive mutations, exon-shuffling, or alternative splicing during mRNA expression. Expression and translational differences of paralogues play a great role in defining the high variability in venom phenotypes [5,7,17], generating the genetic bases for phenotypic diversity [6,18,19,20]. In this context, we have recently demonstrated that some paralogues transcribed in *B. atrox* venom glands were highly expressed and present in high abundance in the venom proteomes of all snakes analyzed. These genes code for isoforms with sequence similarity to functionally important toxins already characterized and conserved in the venom of other species of vipers. We suggested that these isoforms act as generalized core function toxins, in contrast to a greater number of different transcripts in each toxin family that are low and differentially expressed among the individuals, making a greater contribution to venom variability and perhaps coding for adaptive toxins [5].

The variability in venom composition is observed at every taxonomic level in snakes, among individuals of the same species, as well as over the lifetime of a single individual. Variability has been associated to factors such as ontogeny [21,22,23,24], sex [25,26], geographical distribution [27,28,29], snake stress [30], and environmental conditions [31,32]. Many studies attribute diet as the major force driving venom variability [14,33,34,35,36]. Even the ontogenetic variability in venom composition is associated with diet. Diet shifts during ontogeny are ascribed to be the major player for the establishment of mechanisms involved in differential modulation of toxin expression and translation [37,38]. In this regard, Andrade and Abbe [39] compared ontogenetic differences in the efficacy of venoms from two species of *Bothrops* that differ in dietary habits in juvenile or adulthood. The authors observed that venoms from juvenile *Bothrops jararaca* snakes have higher toxicity in anurans than the venom of adult specimens, while venoms of adult specimens are more toxic to mammals, which well correlates the action of venom with dietary habits. On the other hand, shifts in venom toxicity were not observed in *Bothrops alternatus* specimens, which feed on mammals in both juvenile and adult stages. The relationship between diet and venom composition was experimentally tested by Sanz, Gibbs, and colleagues [35,37] who analyzed the proteome of *Sistrurus* spp. snakes fed on different diets (mice, lizards, or frogs) over a 26 month period. The authors reported changes in the relative abundance of major protein families in venom.

It is currently suggested that the extensive snake venom variability may be due to differences in gene expression patterns that may facilitate rapid adaptive response in the face of changing environmental pressures [5,16,17]. However, the plasticity of this phenotype is still uncertain, mostly due to the reduced experimental possibilities of the wild species and technical problems for testing ecological or evolutionary hypothesis. Keeping snakes under controlled conditions is a good experimental situation to test hypotheses explaining venom variability, particularly to investigate the plasticity of the venom phenotype. Captive specimens experience environmental changes, including the food supply, which is usually restricted to a single type of prey, alive or more frequently dead, or even consisting of industrialized animal food. In this aspect, it is reasonable to expect that these drastic changes from natural environments may interfere with snakes kept under captivity, changing the expression patterns of toxin genes and resulting in modifications in venom composition. This hypothesis has been tested recently by different groups and the results are still inconclusive. Some evidence indicates little influence of captivity maintenance on venom composition [26,40,41,42,43], while others show evidence that the environmental changes due to introduction to captivity may modify venom composition [44]. However, these studies compare venoms from groups of snakes recently caught in the wild with groups of other specimens maintained for a long term under captivity. In most of these studies [26,41,42], a high individual variability among venoms from snakes from the same group was observed and the presence of distinct phenotypes within the groups impair any identification of parameters that might have changed under captivity. In a well-controlled study, Gibbs and coworkers [37] evaluated the changes in venom composition in samples obtained by periodic extractions of individual *Sistrurus miliarius barbouri* snakes, accompanied for 26 months after they were introduced to captivity. The authors reported evidence of venom phenotype plasticity in both juvenile and adult *S. m. barbouri* and that, at least in adults, prey consumed apparently influenced the relative abundance of distinct classes of venom proteins. However, most analyses were still conducted using pooled venoms and individual variability in initial samples could have influenced the results. Recently, Rex and collaborators [45] analysed changes in venom composition of sixteen adult *Crotalus atrox* specimens, maintained in the lab for eight months on a controlled diet, and did not observe significant changes in the phenotype. However, the period of the study was short and might not be sufficient to express the alterations induced by the new environment/diet. Moreover, variability was observed, in most cases, in adaptive toxins that are usually subjected to fluctuating expression levels. In this regard, the individual evaluation of core function toxin plasticity over time, in response to environmental and dietary changes, would be an important requirement to generate consistent data to indicate venom variability as an adaptive response.

Here, we analysed the modifications introduced in the composition of venom from 13 specimens of adult *B. atrox* snakes, captured in the wild from different environments and maintained under captivity for a period longer than three years, attempting to identify major modifications in the expression of the core-function venom toxins. *B. atrox* snakes were chosen as they are generalist snakes [46], widespread in the Amazon and very well adapted to different phytogeographical sceneries. Thus, the confinement of the specimens in captivity, under controlled temperature, humidity, light conditions, and feeding strictly with lab mice, represents a great modification in snake living habits when compared to natural conditions. Under such environmental modifications, changes in venom composition are expected and may reflect the plasticity of this phenotypic trait.

## 2. Results

Individual variations in venom composition were analyzed in venom samples periodically extracted from the specimens selected for this study after introduction to captivity. During a period longer than three years, venom samples were extracted from each specimen, in intervals longer than 40 days, as indicated in Supplementary Table S1. Venom composition was analyzed in each individual sample according to the chromatographic profile in reverse-phase C18 HPLC columns (RP-HPLC), which is a well-accepted method to screen and compare venom composition in a large number of samples [37,45,47,48,49]. Additionally, this is the protocol we used to perform proteomic analyses from pools or individual venom samples from *B. atrox* snakes collected in the same areas, in which we characterized the toxins present in each of the RP-HPLC fractions [50], which allows for inferences in venom components present in each chromatographic fraction discussed here.

**Figure 1:**
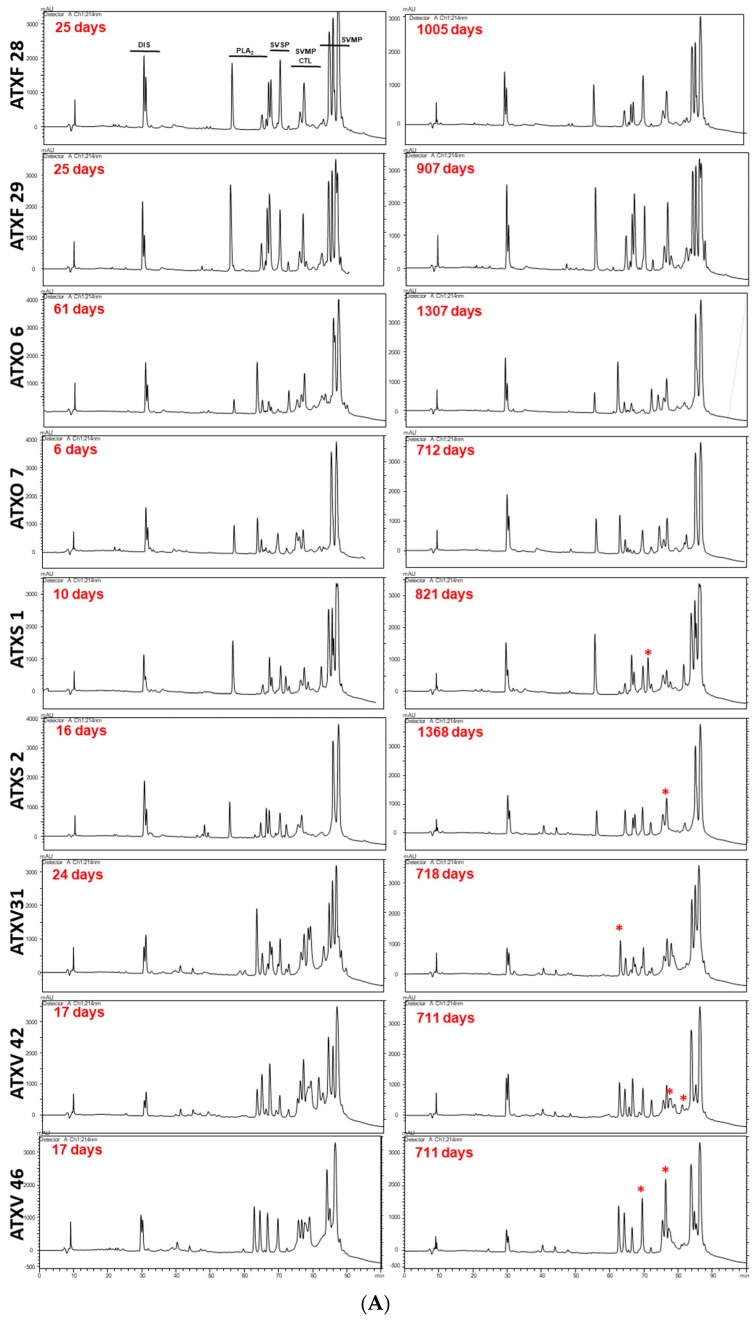

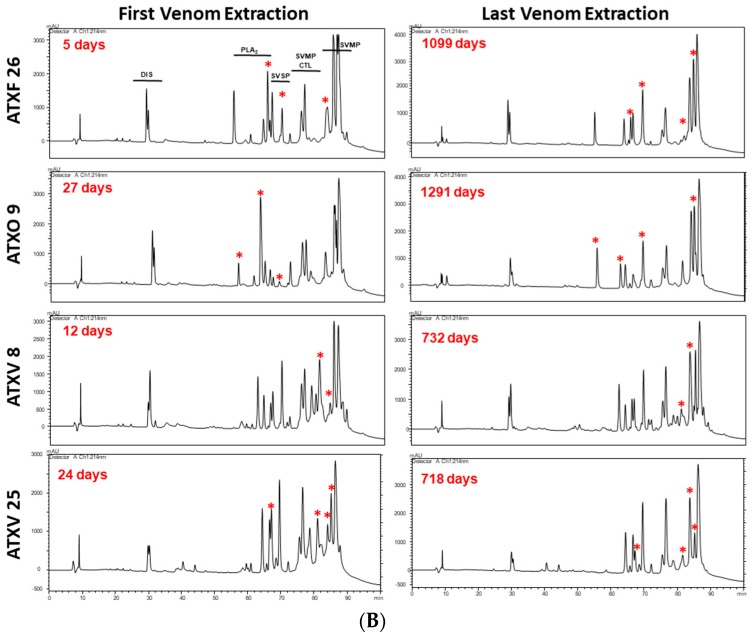
Comparison of the chromatographic profiles of venom samples from each snake, collected just after their capture in the wild (first extraction) and at the longest period they were maintained in captivity (last extraction). Samples containing 5 mg of crude venom were fractionated by RP-HPLC, as described in the Methods section. Regions eluting disintegrins (Dis), phospholipases A_2_ (PLA_2_), serine proteinases (SVSP), C-type lectin-like toxins (CTL), and metalloproteinases (SVMP) are annotated according to their elution pattern observed in a previous study [50]. (*) corresponds to fractions with at least 5% peak height and with fold changes >1.5. (**A**) Venoms with low variability between samples from the first and last venom extractions (**B**) Most variable venoms comparing samples from the first and last venom extractions.

The results shown here support previous evidence of individual variability in the composition of the venoms of *B. atrox* snakes. In Figure 1A,B (left columns), we show the chromatographic profiles of the venom samples extracted from the 13 specimens soon after their capture in the wild (first extraction). The chromatograms follow a general elution profile, as observed in other studies with *Bothrops* snakes [4,50]; however, with marked differences in the heights of several peaks eluted in the disintegrins (Dis), SVSP, CTL, and PLA_2_ regions. In particular, the K-49 eluting fraction (~55 min) shows great variation in peak height among the venoms and is absent in venoms from floodplain snakes (ATXV 8, 25, 31, 42, 46). In the region eluted after 80 min, which concentrates mostly SVMPs, some venoms present two major distinct peaks (ATXF 26, ATXO 6, ATXO 7, ATXO 9, ATXV 8, ATXV 25, ATXV 46, ATXS 2) while the others present a more complex profile with the distinction of three or more peaks (ATXF 28, ATXF 29, ATXV 31, ATXV 42, ATXS 1).

Venom variability was then individually compared with samples extracted in different periods during the time the snakes were kept in captivity. The first venom samples were extracted at the earliest possible time after capture, which ranged from 6 to 25 days, except for one specimen from which first venom extraction occurred 61 days post capture. The last venom extraction depended on the latest possible time to maintain the animals in good health conditions under captivity and ranged from 711 to 1368 days. To follow the characteristics and kinetics of eventual changes in venom composition, we evaluated samples from each specimen extracted at different intervals of time. As several samples repeated the chromatographic profiles of the venom samples collected immediately earlier or at the posterior periods, we selected to present only the chromatograms of samples obtained in the first and last extractions. The chromatograms of all analyzed samples are shown in the Supplementary Chromatographic Data. We considered the ones that presented more than 5% peak height in at least one chromatogram with fold changes higher than 1.5 as variable fractions, as shown in Supplementary Table S2.

The comparison of chromatograms of samples from the first and last venom extractions is shown in Figure 1A,B. Venoms from snakes ATXF 28 and ATXF 29, collected from the forest, and ATXO 6 and ATXO 7, collected from pasture areas, displayed very similar chromatograms (Figure 1A). Only one peak presented an increase in height in venoms ATXS 1 and ATXS 2, collected from savannah, and these peaks represent a low percent of the total area of the chromatogram and were eluted in the regions corresponding to SVSP and CTL [50]. Venom samples from the first and last extraction of snakes collected from the floodplain area (ATXV 31, ATXV 42, and ATXV 46) presented a slightly higher variability in the height of peaks eluted in different points of the chromatographies (Figure 1A), in which the predominant proteins are PLA_2_s, SVSPs, CTLs, and SVMPs [50]. These changes were restricted to less abundant components and the peak height of variable fractions fluctuated in chromatograms corresponding to venom samples of intermediate extractions, indicating an oscillating pattern in their expression, as previously correlated to adaptive toxins [5].

A distinct group composed of four snakes presented drastic changes in the chromatograms of different extractions during the period of the study. These changes occurred in fractions eluting the major peaks that include SVMPs, the core function toxins. In addition, the changes were observed in a specific time interval and persisted in the following extractions. These snakes include one specimen collected from the forest (ATXF 26), one from the pasture (ATXO 9), and two from the floodplain (ATXV 8 and ATXV 25) areas (Figure 1B). In ATXF 26 chromatograms, the initial venom extractions resolved the SVMP eluting region in two predominant peaks, while an extra high peak was eluted at 85.7 min in late extracted venom samples. We also observed an increase in the height of the peak eluted at 69.4 min and a reduction of peaks eluted at 65.1 and 82. 9 min, which correspond to regions eluting PLA_2_, SVSP, SVMP, and CTL toxins. In ATXO 9 venom samples, the peaks eluted in the SVMP region turned from a simpler two-peaks profile, in samples from early extractions, into a three-peaks pattern in venom samples extracted in late periods, with a better definition of a peak eluted at 85.5 min. Another relevant difference was the increase of the peak eluted at 68.4 min, which may correspond to a SVSP that was almost absent in venom samples extracted in initial periods. The chromatograms of ATXV 8 samples also revealed a similar pattern of variability highlighting the differences related to the increase of the peak collected at 83.7 min, at the SVMP-eluting region, with a shift from the two to three-peaks pattern. Reductions in the height of peaks eluted at 78.2 min, 79.5 min, and 80.6 min were also observed and these peaks are likely to contain mostly CTLs and other SVMP isoforms. Chromatograms of the ATXV 25 venom samples revealed a simpler profile and the variability pattern differed from the previous ones. In the SVMP-eluting region, the three-peaks pattern was observed in all samples, however, with an increase of the first peak, eluted at 84.2 min, and a decrease of the peak eluted at 85.2 min. A decrease in the height of the peaks eluted at 67.2 min and 81.1 min, corresponding to SVSPs, PLA_2_s, CTLs, and other SVMP isoforms, was also noted (Figure 1B).

**Figure 2:**
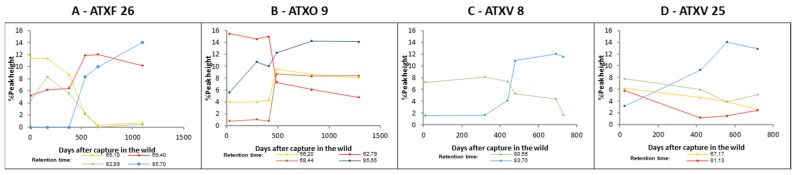
Differential expression patterns of core function toxins of ATXF 26, ATXO 9, ATXV 8, and ATXV 25 snakes during the period of the study. Variations in % peak height of fractions larger than 5% peak height, with fold changes >1.5, in different periods after captive maintenance, according to their retention time.

We next evaluated the kinetics in which these modifications occur. For this purpose, we analyzed the % peak height of the variable fractions in chromatograms of ATXF 26, ATXO 9, ATXV 8, and ATXV 25 venom samples obtained in different periods after introduction to captivity (Figure 2). In ATXF 26 (Figure 2A), the changes in the expression of the core function toxins occurred between 379 and 537 days after introduction into captivity. In ATXO 9 venom samples, the shift occurred after 486 days (Figure 2B), in ATXV 8, after 480 days (Figure 2C), and in ATXV 25, the % peak height did not change so drastically, but occurred mostly after 419 days (Figure 2D).

**Figure 3:**
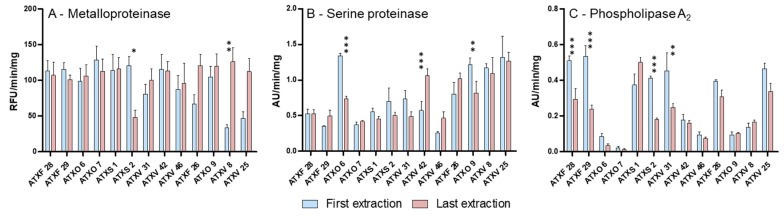
Enzymatic activities of venom samples from the same snake collected just after their capture in the wild (first extraction—blue) and at the longest period they were maintained in captivity (last extraction—roseate): (**A**) Metalloproteinase activity was measured by fluorimetric assays using the Abz-AGLA-EDDnp substrate; (**B**) Serine proteinase activity was calculated by the hydrolysis of Benzoyl-arginine-p-nitroanilide (L-BAPNA); (**C**) Phospholipase A_2_ activity was assayed using the 4-nitro-3- [octanoyloxy] benzoic acid substrate. (*) Significant differences between enzymatic activities of venom samples from first and last extractions of the same snake (* *p* < 0.001; ** *p* < 0.01; *** *p* < 0.05).

Functional variability was then accessed in these venoms according to the enzymatic activity of the following major venom toxin groups: SVMPs, SVSPs, and PLA_2_s (Figure 3). Comparing venom samples from the first and last extractions of the 9 specimens with minor variability, SVMP activity was homogeneous among venom samples, except by a decrease of almost 50% in the last venom extraction of ATXS 2 snake (Figure 3A). Opposed to that, SVSP and PLA_2_ activities varied considerably among venom samples extracted from the different specimens, demonstrating functional differences among the venoms of the individuals included in the study. Comparing venom samples from the first and last extractions, SVSP activity (Figure 3B) differed more significantly in two venom samples (ATXO 6 and ATXV 42). Four samples (ATXF 28, ATXF 29, ATXS 2, and ATXV 31) presented statistically significant changes in PLA_2_ activities (Figure 3C). These changes in enzymatic activities did not correspond to alterations in the peak sizes of chromatographic fractions.

The enzymatic assays of the most variable venoms showed an apparent increase in the metalloproteinase activity in ATXF 26, ATXV 8, and ATXV 25 venoms; however, the differences observed found a statistical support only in ATXV 8 venom samples (Figure 3A). Serine proteinase activity was significantly lower in ATXO 9 samples from the last extraction and no significative differences were observed in phospholipase A_2_ activity of first and last extracted venom samples of these snakes.

## 3. Discussion

Studying the processes that drive snake venom variability is a topic of great importance for attempting to understand how venom complexity relates to fitness of snakes or corresponds to the evolution of different taxa. The study of venom variability also has important medical consequences, as it may elucidate differences in venom toxicity reflecting in pathogenicity and antivenom efficacy in human accidents. Keeping snakes under controlled conditions provides a suitable experimental situation to investigate forces acting on venom variability, particularly for testing the hypothesis of the plasticity of venom phenotypes. Using this approach, we observed important changes in the venom composition of 4 out of 13 snakes included in the study.

Initially, our data confirmed the individual variability in venom chromatographic profiles and enzymatic activities, even in groups collected at the same environment, confirming our previous evidence of venom variability among snakes from the same populations [5]. High individual variability in the relative abundance of the toxins was also observed in previous studies comparing venoms from wild and captive snakes from other *Bothrops* species and was observed among snakes recently caught and also snakes kept in captivity [26,40,41,42]. This is a very intriguing point since it is reasonable to expect that snakes maintained in captivity for long periods, subjected to the same diet, feeding, milking regularity, and stable temperature and humidity conditions would present less variability in the composition of their venoms.

Comparing variations in venom composition of samples collected periodically from each specimen included in this study, we observed that nine snakes presented only small changes in a few chromatographic peaks, indicating that the expression of genes coding for the major toxins followed the same modulations, in spite of the changes in diet or environmental conditions. This minor variability pattern was a little more evident in the venoms from snakes collected from the floodplain area. In previous studies, we reported that pools of venoms from snakes collected from the floodplain habitat differed from the others on functional activities [50,51] and higher individual venom variability was observed among this group, suggesting that a higher variability may be a characteristic favored in snakes inhabiting this type of environment. The major venom-enzymatic activities varied in the nine specimens included in this group and these changes found no correlation to the predicted composition of the fractions whose peaks showed differences in height, suggesting that small fluctuations in the expression of different isoforms of these compounds, recorded due to the high sensitivity of the enzymatic assays, may contribute to this apparent functional variability of the venoms, as reported in other comparative studies using enzyme assays [40,44,52]. Nevertheless, it is important to note that the variability within this group of nine snakes was related to toxins present at low abundance in the venoms.

Opposed to that, venom samples from ATXF 26, ATXO 9, ATXV 8, and ATXV 25 snakes presented marked differences in the core function toxins, considered essential for the venom toxicity [5]. In these venoms, variability was observed in peaks representing fractions collected in the SVMP eluting region, with major changes in the shape and elution time of the major peaks. It is important to point out that these differences in peak resolution were not related to artifacts in the chromatographic procedures since the chromatographic procedures were repeated on different occasions and with different venom samples, resulting in similar profiles. Therefore, these changes suggest that different isoforms of SVMPs are expressed during the period snakes stayed under captivity and these changes are very likely to result in functional variability of the venoms, as previously shown for *B. neuwiedi* SVMP isoforms [3].

The puzzling question is why ATXF 26, ATXO 9, ATXV 8, and ATXV 25 snakes, and only them, presented such changes in SVMP expression profiles. Changes were not directly related to ontogenetic stage, sex, or previous environmental background of the snakes. We also excluded the possibility of interferences by asynchronous venom regeneration, as the intervals between each feeding and venom extraction were longer than 40 days. The venom production cycle peaks between 4 and 9 days after extraction, returning to a quiescent state around 40 days after stimulus [53]. However, it is important to note that two of the variable snakes (ATXV 8 and ATXO 9) were the smallest females when captured from the wild and the changes in the venom may be related to the continuous growth or body conditions of the snakes. Another particular observation was that three of these four snakes (ATXF 26, ATXO 9, and ATXV 8) were collected from the wild in the raining season. During this period the snakes are more active and prey is more diverse, with a greater abundance of amphibians [54]. Thus, one could speculate that snakes collected at this season could be more reactive to the confinement and diet modifications introduced by captivity, explaining the observed modifications in the venom of this group. Interestingly, in these snakes, the changes observed in the venom samples occurred between 400 and 500 days after introduction to captivity. Our first interpretation for that was some uncontrolled occurrence in the serpentarium provoking stress or changes in the snake care. However, this hypothesis was ruled out since these snakes have been introduced to captivity at different moments, in periods two years apart. The other possibility was that 400 days corresponds to an average period for incorporation of the new diet on snake tissues. This hypothesis was tested by Martinez [55] evaluating the *turnover* period of the δ^13^C correspondent to diets available in the natural environment, which are usually based on plants with photosynthetic cycle type C_3_, to the diet used in captivity that include lab mice fed with formulas consisted mostly by plants of photosynthetic cycle type C_4_. The isotopes were measured in blood, scales, and venom and the *turnover* periods of δ^13^C were always under 100 days, also ruling out the correlation between the time of the shift in venom composition to incorporation of the new diet on snake tissues. Interestingly, in a similar study [45], biologically significant changes in venom composition did not occur within individual *C. atrox* specimens as a function of captivity/diet in a period of 8 months, and observed differences occurred in minor venom enzyme components. This is in agreement with our data, showing that only fluctuating quantitative variations restricted to low abundant components were observed in the first period of captivity, which lasted approximately 400 days.

Taken together, our results clearly show that venom composition does change during the life cycle of specimens *B. atrox* snakes, indicating the plasticity of the phenotype, as hypothesized above. The possible mechanisms involved in such occurrence are still unknown, but it is very likely that postgenomic mechanisms may have a major influence on modifications in the composition of snake venoms. Recent reports indicate that chromatin structures and highly expressed transcription factors contribute to the regulation of venom gene expression [56]. Some studies report that microRNAs (miRNAs) may influence the ontogenetic pattern of venom protein translation in rattlesnakes, controlling the expression of the two major core function toxins of adult or juvenile snakes [21,57]. However, it is still unknown what are the external cues driving the switch of the mechanisms and also if other still unknown mechanisms may contribute to the generation of adaptive venom variability.

It is currently assumed that diet controls the natural selection of toxin genes for a certain taxa [58] and there is much evidence for the arms race between snakes and their predators [59,60] or prey [61], showing the strength of diet in the natural selection of toxin genes. Diet was also correlated to plasticity of venoms in adult snakes [14]. However, some recent studies [31,32] reported that diet composition could not explain the venom variation of *Crotalus scutulatus* snakes. Instead, venom divergence would be strongly correlated with environmental conditions. In this study, we reinforce the last assumption, as we did not find evidence that could attribute the observed venom variation in certain snakes to the change in diet, but we showed clear examples of changes in venom composition during the lifetime of adult *B. atrox* snakes, which provide some of the first evidence for plasticity in the core function of snake venom phenotypes. However, causes and consequences involved in the conservation/variation of the venom phenotype, or how observed changes could correlate to venom functions, still remain as puzzles to be solved.

## 4. Materials and Methods

### 4.1. Snakes, Venoms and Captivity Conditions

Thirteen *B. atrox* adult snakes were collected in the western part of the State of Pará, Brazil, under ICMBio/SISBio license 32098-1 and SISGEN number A78BD88. Three specimens were collected in Floresta Nacional do Tapajós, a National Forest located next to the Tapajós River, about 80 km south of the Amazon River, in the municipality of Belterra; three specimens were collected in a pasture area in the municipality of Oriximiná, on the north shore of the Amazon River; two specimens were collected in a savannah area in the east shore of Tapajós River, about 20 km south of the Amazon River, in the municipality of Santarém; and five specimens were collected in a floodplain area of the Amazon River, in the municipality of Santarém. After capture, the snakes were transferred to the Herpetarium of Laboratório de Pesquisas Zoológicas, Centro Universitário da Amazônia (UNAMA), in Santarém. The snakes were cataloged in a database containing the code of each animal, the geographical coordinate of the collection site (GPS), the date, the sex, and the snout-vent length (SVL). The snakes were kept in plastic boxes at a temperature of approximately 25 °C, in a light/dark 12 h cycle, with water ad libitum and controlled feed, restricted to live lab mice weighing up to 15% of the weight of the snake, offered every 40 days. Venom samples were collected using manual extraction techniques, in different time intervals of approximately 40 days or longer periods, always one week before feeding the snakes. After extraction, venom samples were freeze-dried and kept at −20 °C until used. Animal care and procedures used in the handling of snakes were in accordance with the guidelines of the Ethical Committee for Animal Research of Instituto Butantan (1244/14). Data of snake characteristics and the dates of venom extractions are listed in Supplementary Table S1.

### 4.2. Venom Fractionation by Reverse Phase Chromatography

Individual *B. atrox* venoms were fractionated using reversed-phase high-performance liquid chromatography (RP-HPLC) following previously described methods [4]. Briefly, 5 mg of crude lyophilized venom was dissolved in 250 μL of 0.1% trifluoroacetic acid (TFA) and injected onto a Vydac C18 column (250 mm × 4.6 mm, 10 µm particle size) coupled to a Shimadzu LC 20-AT HPLC system. Proteins were eluted at 2 mL/min with a gradient of 0.1% TFA in water (solution A) and acetonitrile (solution B) (5% B for 5 min, 5–15% B over 10 min, 15–45% B over 60 min, 45–70% B over 10 min, 70–100% B over 5 min, and 100% B for 10 min). The fractionation was monitored at 214 nm. The venom profiles of samples collected at the first venom extraction (TI) and samples extracted in periods longer than 2 years were compared individually, with respect to retention times and the height of eluted peaks. Variable fractions had their composition predicted according to peak shape and retention of a previous comprehensive proteomic analysis of *B. atrox* venom from snakes collected in the same areas in which the major HPLC chromatographic peaks had their components identified by mass spectrometry [50] and corresponded to the elution profiles of the major venom protein families described from venoms of viper snakes [47].

### 4.3. Enzymatic Assays

Metalloproteinase, serine proteinase, and phospholipase A_2_ activities were assayed as previously described [62]. For metalloproteinase assays, venom samples (10 µg) were incubated with FRET (Fluorescence Resonance Energy Transfer) substrate Abz-AGLA-EDDnp (GenOne Biotechnologies) and the enzymatic reactions were monitored in a SpectraMax^®^ M2 fluorimeter (Molecular Devices), with excitation at 320 nm and emission at 420 nm, at 37 °C in kinetic mode over 5 min, and with a read range of 1 min. The results were expressed in RFU/min/µg. Serine proteinase activity was assayed using the chromogenic substrate benzoyl-arginyl-p-nitroanilide (L-BAPNA) (Sigma-Aldrich^®^), incubated with venom samples (50 µg) at 37 °C for 40 min. Hydrolysis was measured spectrophotometrically at 405 nm and activity was expressed as absorbance at 405 nm/min/mg of venom. Phosphilipase A_2_ activity of venom samples (20 µg) was assayed using a substrate 4-nitro-3- [octanoyloxy] benzoic acid (Enzo^®^ Life Sciences) at a final concentration of 320 µM, incubated for 40 min at 37 °C, and activity was determined according to the absorbance at 425 nm and expressed as absorbance/min/mg of venom. The results represent the mean ± SD of three independent experiments. Statistical analysis was carried out by one-way ANOVA analysis of variance, followed by Dunns post-test or, for paired data, by two-way ANOVA followed by a Bonferroni post-test. The significance level was considered if *p* < 0.05.

## Supplementary Materials

The following are available online at https://www.mdpi.com/2072-6651/11/5/294/s1, Supplementary Table S1: General information on the snakes included in the study and venom extraction dates; Supplementary Table S2: Values of area and height of variable peaks between first and last venom extractions; Supplementary Chromatographic Data: Details of chromatograms of all samples analyzed in the study, included or not in the manuscript.
